# Gastric Teratoma: A Rare Neoplasm

**Published:** 2012-04-01

**Authors:** Mahavir Singh, KN Rattan, YS Kadian, Neha Hasija

**Affiliations:** Department of Paediatric Surgery, Pt.BD Sharma, Postgraduate Institute of Medical Sciences Rohtak; 1 Department of Anaesthesiology, Pt.BD Sharma, Postgraduate Institute of Medical Sciences Rohtak.

**Keywords:** gastric teratoma, male, neonate

## Abstract

A 3-day-old male child was brought to the hospital with complaints of abdominal distension and a mass in the upper abdomen causing respiratory difficulty. Child underwent exploratory laparotomy and a large multicystic mass arising from postero-inferior wall of the stomach along its greater curvature was excised and stomach repaired. On histopathology it came out mature gastric teratoma.

## INTRODUCTION

Gastric teratoma is an extremely rare neoplasm, accounting for less than 1% of all teratomas occurring in infancy and childhood [1]. The first case of gastric teratoma was reported in 1922 by Eustermann and Sentry, since than only 102 cases have been reported in the literature [2]. They usually present as a palpable mass in the epigastrium and left abdomen with abdominal distension. Because of rarity of the tumor, we are reporting this case with review of literature.

## CASE REPORT

A 3-day-old male child (weight 2.9 kg) was brought to the hospital with complaints of abdominal distension and a mass in the upper abdomen noticed by them just after birth. Patient was in respiratory distress because of large size of tumour. The child was born full term by normal vaginal delivery and cried immediately after birth. The patient tolerated breast feeds without any vomiting. There was no history of exposure to any drugs or radiation to the mother in the antenatal period. Initial abdominal examination showed fullness in the left upper abdomen and a large, irregular firm mass at the left hypochondrium and epigastric region leading to massive abdominal distension.

Hematological profile revealed hemoglobin of 15.0 g/dL and total leukocyte count of 14,500/cm3. Kidney function tests, liver function tests were within normal limits. X-ray abdomen showed a mass effect in the left hypochondrium with diffuse calcifications. Ultrasonography revealed a multicystic mass with mixed echogenicity in left upper abdomen, origin of which could not be appreciated, displacing the left kidney downwards. There was no free fluid in the peritoneal cavity. Computed tomography demonstrated a large lobulated heterogeneous mass with solid and cystic components with calcification and inferiorly reaching upto pelvis, displacing gut loops (Fig 1).

**Figure:**
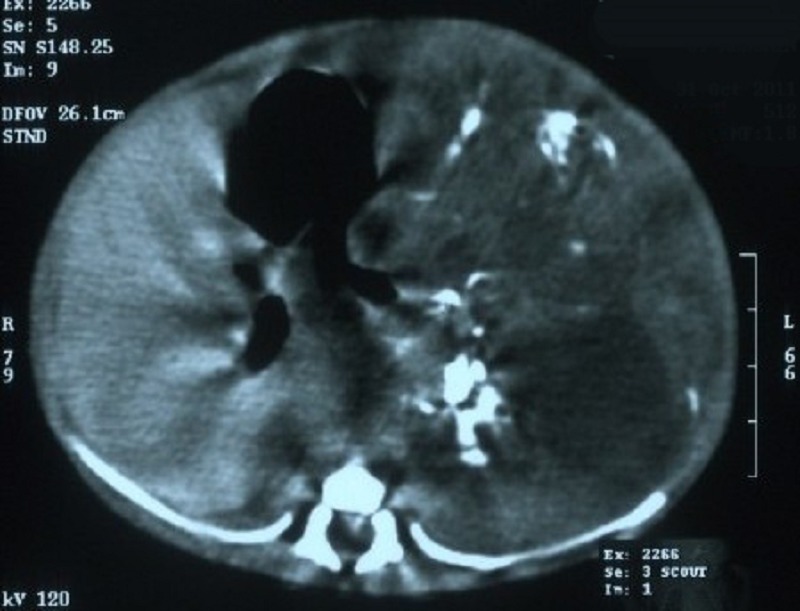
Figure 1: CT scan of the abdomen demonstrated a large lobulated heterogeneous mass with solid and cystic components with calcification.

The child was taken up for surgery. Exploratory laparotomy revealed a large multicystic mass arising from postero-inferior wall of the stomach along its greater curvature (Fig. 2). Greater part of the mass was lying outside the stomach while a small part was extending into the lumen of the stomach. The mass was excised in toto with a small fringe of the gastric wall from which the lesion originated. The stomach was repaired in layers. The excised specimen measured 13x10x8 cm. Cut surface revealed large cystic areas with solid areas. Solid tissue was composed of greyish white areas with foci of cartilage and bone (Fig. 3). Histopathology report was mature gastric teratoma.

**Figure:**
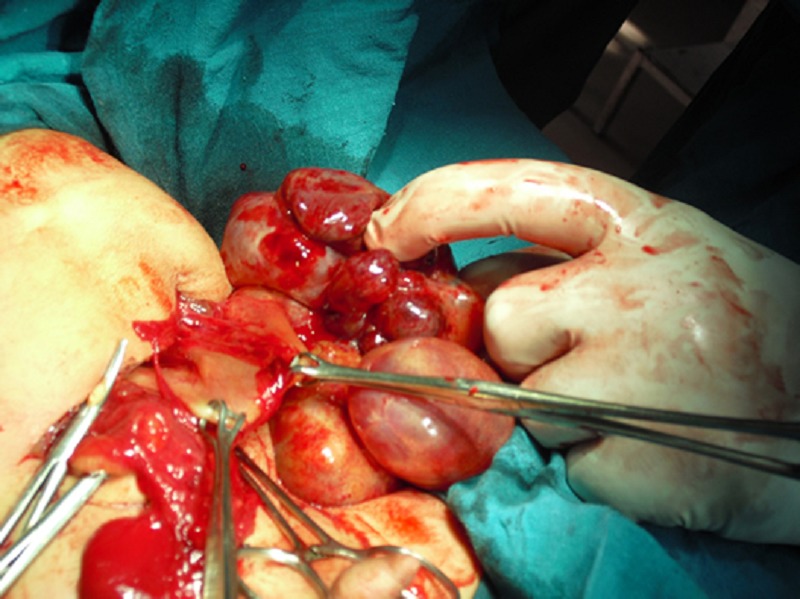
Figure 2: Intra-operative photograph showing a large multicystic mass arising from postero-inferior wall of the stomach along its greater curvature.

**Figure:**
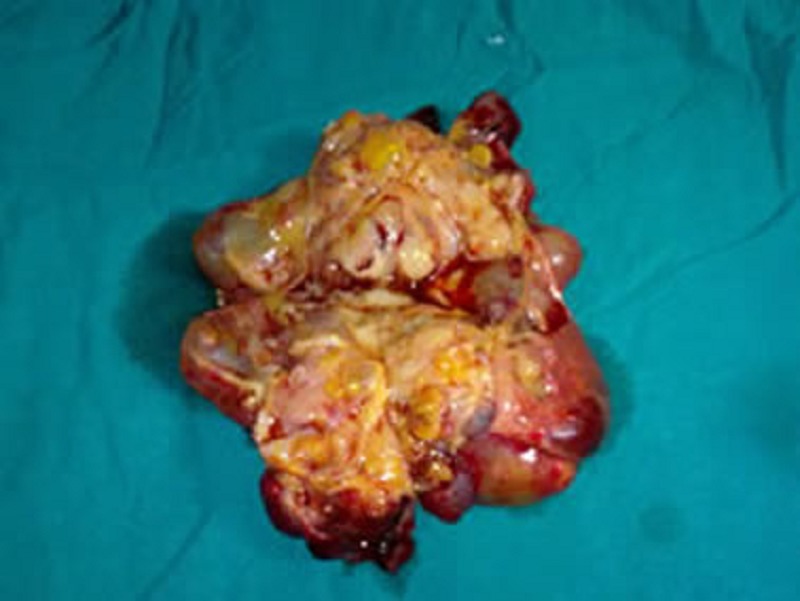
Figure 3: Cut section of the mass.

## DISCUSSION

The most common site of teratoma in infancy and childhood is sacrococcygeal teratoma (60-65%), gonadal (10-20%), mediastinal (5-10%), presacral (5%) and rarely intracranial, retroperitoneal and cervical [1].

Gastric teratomas can be mature and immature based on the presence and differentiation of neuroglial tissue. Mature gastric teratomas contain mature glial tissue along with other derivatives of all germinal layers. Mature gastric teratomas are considered benign tumours, whereas, the malignant potential is present in immature gastric teratoma. The term ‘malignant teratoma’ is restricted to the endodermal sinus tumor (EST) or yolk sac tumor and choriocarcinoma. Almost all reported gastric teratomas have been mature and benign with only two cases of malignant transformation reported so far [3].

Majority of gastric teratomas are exophytic (>60%), endophytic growths are present in about 30% of cases, mixed exophytic and endophytic growths are present in only few cases. Although gastric teratomas can arise from any part of the stomach, common sites are the lesser curvature of stomach, antrum and fundus of stomach along the posterior wall. Some of these tumours are pedunculated [4,5].

These large tumours presenting in the newborn may cause premature labor or dystocia. Respiratory difficulty is also common caused by upward displacement of the diaphragm by the tumour. Some infants have vomiting, haematemesis or malena especially those with endogastric component causing ulceration of overlying mucosa [6].

In most of the cases the preoperative diagnosis of gastric teratomas is difficult. Abdominal radiograph, ultrasonography, CT/MRI and endoscopy are important diagnostic tools. Plain films usually reveal a soft tissue mass with associated irregular areas of calcifications. US demonstrate a heterogeneous mass with mixed echogenicity. CT scan demonstrates a mass with solid and cystic components and internal calcifications and fat. CT scan is the modality of choice. When combined with intravenous and oral contrasts, they can detect the origin of the tumour, its relation with gastrointestinal tract and major blood vessels, presence of bones and calcifications, and tumour extent and presence of metastasis. Other modalities like barium meal and gastroscopy has a limited role in the diagnosis of gastric teratoma [7].

Surgical excision is the treatment of choice. Depending upon the extent of tumour partial, subtotal and total gastrectomy has been done. Prognosis following surgical excision has been excellent with report of one case of recurrence [8].

## Footnotes

**Source of Support:** Nil

**Conflict of Interest:** None declared

